# A shift of brain network hub after spinal cord injury

**DOI:** 10.3389/fnmol.2023.1245902

**Published:** 2023-10-17

**Authors:** Kohei Matsubayashi, Munehisa Shinozaki, Junichi Hata, Yuji Komaki, Narihito Nagoshi, Osahiko Tsuji, Kanehiro Fujiyoshi, Masaya Nakamura, Hideyuki Okano

**Affiliations:** ^1^Department of Orthopaedic Surgery, School of Medicine, Keio University, Tokyo, Japan; ^2^Department of Physiology, School of Medicine, Keio University, Tokyo, Japan; ^3^Graduate School of Human Health Sciences, Tokyo Metropolitan University, Tokyo, Japan; ^4^Live Animal Imaging Center, Central Institute for Experimental Animals, Kanagawa, Japan; ^5^Department of Orthopaedic Surgery, Murayama Medical Center (NHO), Tokyo, Japan

**Keywords:** spinal cord injury, network analysis, corticospinal tract, motor function, brain network hubs, resting-state functional MRI, (rS-fMRI)

## Abstract

**Background:**

Spinal cord injury (SCI) causes severe sequelae and significant social loss, depending on the extent of the damage. Most previous studies have focused on the pathology of the spinal cord to develop treatments for SCI. However, it is now known that the brain, which is not directly damaged, also undergoes morphological changes after spinal cord injury, which could affect natural recovery and treatment. In recent years, magnetic resonance imaging (MRI) has been developed to analyze functional changes in the brain. Resting-state functional MRI (rsfMRI), which captures brain activity at rest, can calculate functional connections between brain areas and identify central hubs by network analysis.

**Purpose:**

We aim to investigate functional connectivity in the brain using rsfMRI after SCI and to determine how brain-network main hubs change over time.

**Methods:**

We evaluated rsfMRI in 10 mice of the contusional SCI model and calculated connectivity using graph theory. We evaluated “centrality,” a representative parameter of network analysis. The subtype of centrality was degree centrality, which indicates the hub function of a single area. The five times of rsfMRI were performed in each individual mouse: before injury and at 1, 3, 7, and 14 weeks post-injury.

**Results:**

Before the injury, the degree centralities of the primary and secondary motor cortex were high, suggesting that these motor cortices served as main hubs for motor function. After SCI, the hub function of the motor cortices decreased by 14 weeks. In contrast, hub function in the external capsule and the putamen comparatively increased with time after injury, suggesting that the extrapyramidal/subcortical system, which runs the ventral side of the spinal cord and remains after injury in this model, becomes dominant.

**Conclusion:**

We demonstrated the shift of the brain network hub after SCI. The results of this study provide basic information for understanding brain network changes after SCI and would be useful for treatment selection and evaluation of its efficacy in SCI patients.

## Introduction

Spinal cord injury (SCI) causes dysfunction of the spinal cord at and below the level of injury, followed by impairments such as motor and sensory paralysis. Depending on the extent of the injury, spontaneous recovery is limited and often results in serious sequelae. Worldwide, one million people are affected by SCI each year, yielding a significant social loss (GBD 2016 Traumatic Brain Injury and Spinal Cord Injury Collaborators, 2019). To date, many researches have been conducted to develop treatments for SCI, and most of them have focused on the pathophysiology of the spinal cord itself. Meanwhile, it is known that there are morphological changes in the brain, which is not directly affected after SCI, and it is suggested that this hinders natural recovery and treatment (Jurkiewicz et al., 2006; Wrigley et al., 2009).

Neuromodulation and neurofeedback have been reported to treat brain or spinal cord disease by utilizing the plasticity of the brain. While some approaches to brain diseases are based on the reinforcement of residual brain activity on the affected side (Lorach et al., 2023), there are also treatments based on the theory of interhemispheric inhibition (Ferbert et al., 1992; Hummel and Cohen, 2006), in which interventions are performed on the unaffected side of the brain. Hence, it is necessary to correctly understand the changes in the brain in order to develop a brain-mediated treatment strategy.

Recent advances in magnetic resonance imaging (MRI) have established the methods to analyze brain activity and function. In particular, resting-state functional MRI (rs-fMRI), which captures brain activity at rest, has been developed to analyze functional relationships between different brain regions (Margulies et al., 2010; Lv et al., 2018). Changes in the relations between brain regions have been also reported in SCI studies (Huynh et al., 2019; Zhang et al., 2022), and several approaches are used in rsfMRI to calculate parameters. Among them, graph theory allows for the assessment of connections between hundreds of “nodes” based on functional brain localization. Graph theory analysis typically includes an overall measure such as small-worldness, or individual connectivity, which indicates the strength of connections between each “node.” However, by setting thresholds for each connectivity, traditional “network analysis” can be adapted to reveal the character of the brain network as an unweighted graph.

In this study, we evaluated rsfMRI using a mouse model of SCI to assess “centrality,” a typical network analysis parameter. We focused on degree centrality as a subtype of centrality, which represents the hub function of each node. Imaging was performed before and at 1, 3, 7, and 14 weeks after injury to observe how the major hubs of the brain changed after injury. Focusing on the primary motor cortex, we observed how motor-related hub function shifts after SCI. This is the first study to assess changes in degree centrality in a contusion injury model using longitudinal rsfMRI in mice. When targeting the brain for SCI treatments, brain activity is supposed to change before the symptoms change. Therefore, it is possible that rsfMRI reveals live brain activity which is more sensitive to treatment effects than physical phenotypes such as body function. The results of this study will provide basic information of brain network changes after SCI.

## Materials and methods

### Surgical procedures

Fourteen female C57BL/6J mice (8 weeks old, weighing 18–20 g, Clair Japan Inc) were used in this study. Surgical procedures are described previously (Matsubayashi et al., 2018). Mice were deeply anesthetized with a combination of ketamine (100 mg/kg) and xylazine (10 mg/kg) and fixed with a stereotaxic instrument. An incision was made to expose the surface of the skull. The surface was covered with a thin layer of dental cement using a brush-up technique (Superbond C&B, Sun Medical, Shiga, Japan). An acrylic head bar (3 × 3 × 27 mm^3^) was attached to the sagittal suture of the skull using dental cement. Mice were fed a high-energy diet. After the habituation and the first rs-fMRI imaging described below, mice were subjected to contusional incomplete spinal cord injury. Anesthetized with ketamine (100 mg/kg) and xylazine (10 mg/kg), the dura was exposed after laminectomy at the level of the T9/10 vertebrae. An IH impactor (Precision Systems and Instrumentation, United States, 60 kDyn) was used to induce incomplete contusional SCI at the level of the 10th thoracic vertebra, as described previously (Kamata et al., 2021; Shibata et al., 2021; Kitagawa et al., 2022). Mice for locomotor function underwent only the SCI treatment, and locomotor function was observed for 8 weeks. All animal experimental procedures were performed in accordance with the Laboratory Animal Welfare Act and the Guide for the Care and Use of Laboratory Animals (National Institutes of Health, Bethesda, MD, USA). All experiments were approved by the Animal Study/Committee of the Central Institute for Experimental Animals (approval number: 14078).

### rs-fMRI in awake mice

Mice were habituated for 1 week each time before rs-fMRI, rs-fMRI was performed before and at 1, 3, 7, and 14 weeks post injury (wpi) as previously described (Ratering et al., 2008; Baltes et al., 2009; Basbaum et al., 2009; Bosshard et al., 2012; Komaki et al., 2016; Yoshida et al., 2016; Matsubayashi et al., 2018). rs-fMRI was performed on a 7.0 Tesla MRI system equipped with active shield gradient at a maximum intensity of 700 mT/m (Biospec; 70/16 Bruker BioSpin, Ettlingen, Germany) with a cryogenic orthogonal radiofrequency (RF) surface probe (CryoProbe; Bruker BioSpin AG, Fällanden, Switzerland) for increased sensitivity. The scan protocol used for rs-fMRI was as previously reported. High-resolution T2-weighted images (T2WI) of the whole brain were acquired using the Rapid Acquisition with Relaxation Enhancement (RARE) method with the following parameters: effective echo time (TE); 48 ms, repetition time (TR); 6100 ms, a RARE factor; 8, number of averages; 4, spatial resolution; 75 × 75 × 300 μm^3^, and number of slices; 52. The BOLD fMRI signals were acquired using gradient echo-planar imaging with the following parameters: TE; 20 ms, TR; 1,000 ms, fip angle; 55°, number of averages; 1, spatial resolution; 200 × 200 × 500 μm^3^, and number of slices; 16. Scans were repeated 600 times in 10 min.

### Data analysis

rs-fMRI data analysis was performed as previously reported (Matsubayashi et al., 2018). Briefly, we used SPM12 (Wellcome Trust Centre for Neuroimaging, UCL Institute of Neurology, London, UK), and tailored software for MATLAB was used to adjust slice acquisition timing, motion correction, and registration of different mouse brains to C57BL/6J mouse 3D MRI brain atlas. Normalized functional images were smoothed with a 0.6 mm full width at half maximum filter. Functional connectivity analysis was performed using CONN, with temporal bandpass future applied in the range of 0.009 Hz to 0.1 Hz. Functional coupling between areas was defined as “edges” and analyzed using the regions defined in The Mouse Brain in Stereotaxic Coordinates (Paxinos and Franklin, 2019), where 244 functional areas “nodes” were set. Graph theory-measures were evaluated on raw and threshold networks. All graph theory indices were computed using R software and functions from the igrapgh library.^1^ “Strength” of the whole brain was obtained by adding all the functional connectivity. “Density,” defined as the percentage of possible connections present in a network, is a measure of the overall level of connectivity. To characterize centrality of network analysis, “degree centrality” of each node was calculated, where it corresponds to the number of incoming connections to a single node (Wheeler et al., 2015; Mareckova et al., 2022). To perform network analysis, a threshold value of 0.10 was used for all period data, and correlations that could withstand the threshold were considered connected. Data from a previously reported complete transection SCI animal model was introduced into the study for comparison with the extent of tissue damage (Matsubayashi et al., 2018). Since the previous data had been adjusted according to Allen’s atlas, the corresponding locations were identified according to previous literature (Chon et al., 2019). The centrality of each node was calculated. The three-dimensional configuration was visualized using Processing, version 4.^2^

### Statistical analysis

All data except a supplement are presented as mean ± SEM. A supplement is presented as mean ± SD. Comparisons within the same model group (pre-injury vs. other time-points) were made by paired *t*-tests. Repeated measure ANOVA was used for comparisons between incomplete contusional SCI and transection-complete SCI.

## Results

### The overall network is temporarily increased after SCI

**Figure 1:**
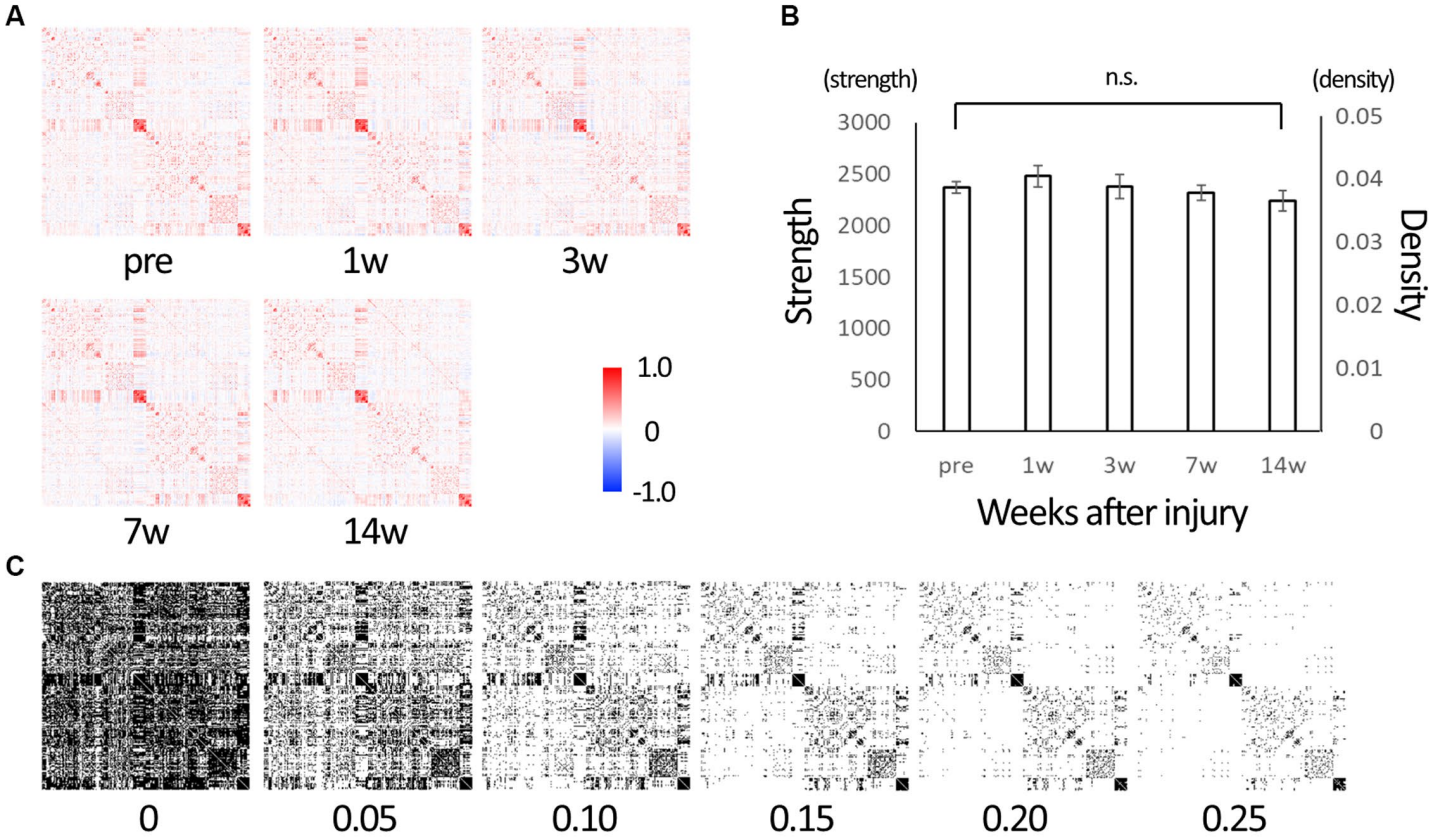
The matrix representing edges between nodes of the whole brain. **(A)** Matrix representing edges (connections) between 244 nodes of the whole brain. Those are of before injury (“pre”), 1, 3, 7, and 14 weeks post-injury (wpi) states. The lower left and upper right are mirror images of each other. Each connection takes a value between −1 (strong negative correlation, blue) and 1 (strong positive correlation, red). **(B)** Strength is the sum of the connectivity of all edges, and density is the average value of the connectivities. After injury, strength tended to increase from the baseline (“pre”), indicating that the brain network is being reorganized, and it decreases over time, indicating the reorganization is settled down. **(C)** Serial data from −1 to 1 were converted to binary data (0 or 1) using various threshold values for use in network analysis. We used 0.10 as the threshold value in subsequent network analysis with reference to the density value (Error bars are shown in SEM for all figures. n.s.: no significant differences).

Graph theory analysis calculated 29,646 inter-node connectivities for 244 whole-brain (122 per hemisphere) regions (Supplementary Table 1). Each connectivity ranged from −1 to 1. The matrices of the connectivities in all time points are shown in Figure 1A. Connectivity was dominated by positive values. The sum of all connectivities was calculated as strength and divided by the number of connections between nodes to calculate density. Figure 1B shows their change over time. Strength tended to increase at 1w after SCI, suggesting that the brain network was disrupted and reorganized. It then decreased over time and was lower than before the injury at 14 wpi, suggesting that a limited network was gradually formed in 14 weeks. To perform classical network analysis, we set a threshold of 0.10 for connectivity and converted serial data to binary data (Figure 1C).

### The network change subsides over time

**Figure 2:**
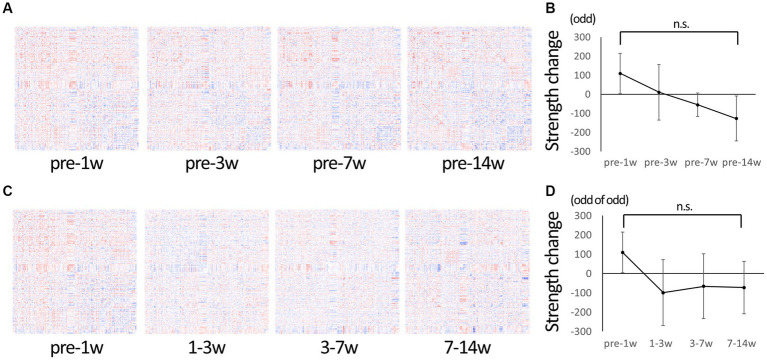
Change in connectivity over time. **(A)** To observe the change of connectivities in detail, the difference from pre-injury (before injury) was calculated for the serial data and represented in matrices. Red indicates increased edges, and blue indicates decreased edges. Increased edges predominate at 1 wpi and decreasing edges predominate at 14 wpi. **(B)** A graph showing changes in strength compared to the pre-injury state. The strength tends to be higher than the pre-injury state at 1 wpi, and it gradually decreased to the lowest value at 14 wpi. **(C)** The difference from the previous time-point was calculated for the serial data and represented in matrices. **(D)** A graph showing the change in strength from the previous time point; it is negative (decreasing trend) at 3–7 wpi and 7–14 wpi, but not larger than the difference of 1–3 wpi, suggesting that the overall change is getting smaller over time (Error bars are shown in SEM for all figures. n.s.: no significant differences).

Serial data were used to evaluate the overall change over time in detail. Figure 2A shows a matrix of changes at each time period compared to pre-injury. The change of connectivity was different depending on the edges. Strength, the total sum of connectivity, increased rapidly in 1 wpi and remained higher than pre-injury in 3 wpi, while it decreased in 7 wpi and further decreased in 14 wpi (Figure 2B). Figure 2C shows the matrix of changes from the previous period: from 1 to 3 wpi showed the greatest decrease in strength, but from 3 to 7 wpi and 7 to 14 wpi the changes became smaller, indicating that the pathological condition of the brain network was settling down (Figure 2D).

### Hub functionality is reduced overall after SCI

**Figure 3:**
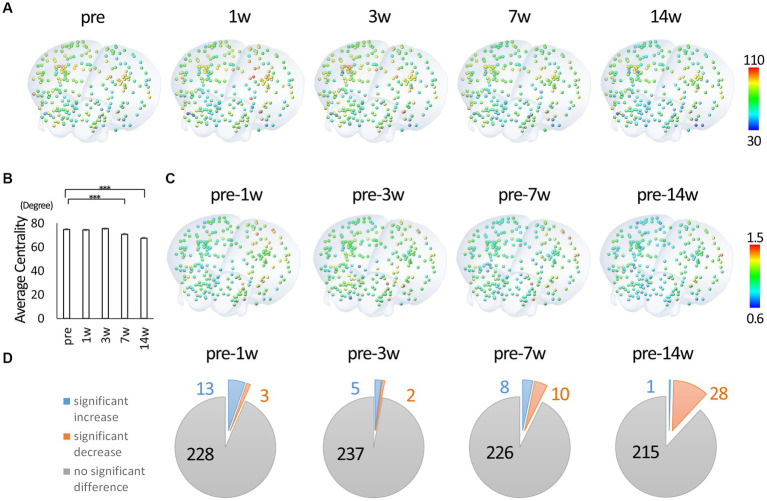
Degree centrality of each node. **(A)** Three-dimensionally reconstructed images of each node colored according to the degree centrality and located by the xyz coordinates. The value 0.10 was used as the threshold for calculating degree centrality from binary data. Degree centrality shows the number of edges of each node and represents the hub function of the network. The highest and lowest values were extracted from all time points and were fitted to a HSV lookup table from 0 to 243 and displayed in a continuous color from blue to red (red indicates nodes with higher degree centrality). **(B)** The change in mean centrality for all nodes. There was a significant decrease in centrality at 7 and 14 wpi compared to the pre-injury. **(C)** Three-dimensionally reconstructed images of each node colored according to the change from the pre-injury. **(D)** The statistical results compared to the pre-injury data. At 1w after injury, some nodes show a significant increase in centrality compared to pre-injury, while the number of nodes showing a significant decline in centrality increases with time (3D overlay image is from atlas.brain-map.org. Error bars are shown in SEM for all figures. ***p < 0.001).

Using the binary data, we calculated degree centrality, a representative network analysis. The degree centrality of each node represents the number of edges connecting the node and indicates hub function. Hence the potential value ranges from 0 to 243 in this study. Figure 3A shows oblique views of three-dimensional reconstruction based on the coordinates in the atlas, with nodes colored with an HSV lookup table from 0 to 243. See Supplementary Figure 1 for view directions and Supplementary Movie 1 for all view angles of reconstructed images. The actual mean value for each node ranged between 30 and 110. There was an overall decrease in centrality through the 14th week post-injury. Figure 3B shows the change in mean centrality for all nodes. In particular, there was a significant decrease in centrality at 14 weeks post-injury compared to each time point, including pre-injury. Figure 3C shows colored nodes with a ratio of 0.6 to 1.5, with the rate of change calculated with the pre-injury data as 1, for comparison with the pre-injury data. Figure 3D shows the statistical results compared to the pre-injury data demonstrating the number of nodes with significant differences. At 1w after injury, some nodes show a significant increase in centrality compared to pre-injury, while the number of nodes showing a significant decrease in centrality increases with time. Overall, it was found that the hub function of each node tended to decrease after SCI.

### Hubs of motor function shift from cortical to subcortical area

**Figure 4:**
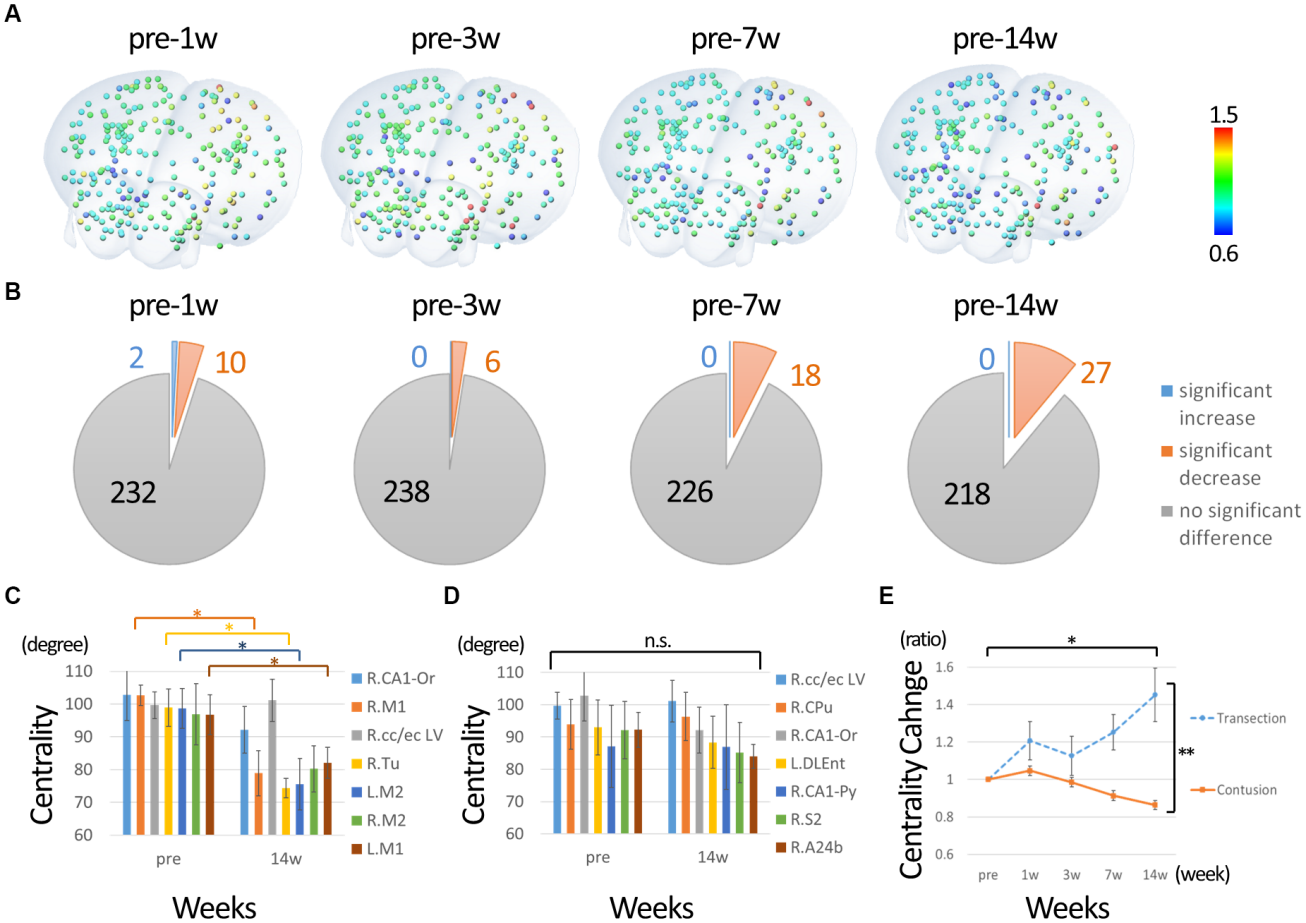
Change in degree centrality of nodes of motor function. **(A)** The percentage change from before the injury was calculated. The nodes colored with ratios ranging from 0.6 to 1.5. **(B)** Shows the statistical results compared to pre-injury. In contrast to the overall analysis, many nodes show a significant decrease in centrality from 1w after injury, with few nodes showing an increase. **(C)** The change in nodes that had high centrality before injury. The highest centrality was seen in the primary and secondary motor cortices, which showed a predominant decrease at 14 weeks post-injury. **(D)** The changes in the nodes that showed high centrality at 14 weeks post-injury. The centrality of these nodes did not increase, but did not decrease, compared to pre-injury, suggesting that they took on a new main hub function in the overall decline. In particular, the centrality of the putamen and corpus callosum/external capsule was relatively high both before and after injury. Please refer to the Supplementary Figure 2 for all time points. **(E)** The changes in centrality of sensorimotor cortex in the completely injured model for comparison with the current incomplete injury. The nodes of sensorimotor region were analyzed instead of primary motor cortex because the position was slightly different in the Paxson and Allen atlases. Compared to pre-injury, the centrality of the sensorimotor cortex decreased in the incomplete injure model, whereas it increased in the complete injury model (R, right; L, left; CA1-Or, CA1 oriens; CA1-Py, CA1 pyramidale; M1, primary motor cortex; M2, secondary motor cortex; S2, secondary sensorimotor cortex; cc/ec, corpus callosum/external capsule; Tu, olfactory tubercle; Cpu, putamen; A24b, cingulate cortex area A24b; DLEnt, Dorsolateral entorhinal cortex. Error bars are shown in SEM for all figures. *p < 0.05, **p < 0.01).

Next, we performed a network analysis focusing on motor function. We extracted nodes that had connectivities to either the left or right primary motor cortex before the injury as motor-related nodes. Only nodes whose connectivity with the primary motor cortex exceeded the threshold (0.1) in each individual were used to calculate centrality. Since no node was below the threshold for connectivity with the primary motor cortex in all individuals (nodes with subthreshold binding to the primary motor cortex were present in some individuals), 244 nodes were included in the analysis. The percentage change from before the injury was calculated. Figure 4A shows the nodes colored with ratios ranging from 0.6 to 1.5. Figure 4B shows the statistical results compared to pre-injury. In contrast to the overall analysis, many nodes show a significant decrease in centrality from 1w after injury, with few nodes showing an increase. Figure 4C shows the change in nodes that had high centrality before injury (approximately 2 SD above average). The highest centrality was seen in the primary and secondary motor cortices, which showed a predominant decrease at 14 weeks post-injury, suggesting that those hub functions were impaired. Figure 4D shows the changes in the nodes that showed high centrality at 14 weeks post-injury. The centrality of these nodes did not increase, but did not decrease, compared to pre-injury, suggesting that they comparatively took on a new main hub function in the overall decline. In particular, the centrality of the putamen and corpus callosum/external capsule was relatively high both before and after injury. Figure 4E shows the changes in centrality in the completely injured model for comparison with the current incomplete injury. Compared to pre-injury, the centrality of the sensorimotor cortex decreased in the incomplete injure model, whereas it increased predominantly in the complete injury model.

## Discussion

In this study, we investigated longitudinal changes in brain network structure after SCI using rs-fMRI analysis in mice. Compared to intact mice (pre- injury), spinal cord-injured mice showed significant changes in the brain network. Graph theory and network analysis revealed that degree centrality, which represents hub function, was altered in motor-related brain regions. These results suggest that network analysis of rs-fMRI can reflect the pathophysiology of neural systems in SCI mice.

Recently, studies using rs-fMRI in SCI have increased. There are many reports in humans (Choe et al., 2013; Hou et al., 2014, 2016; Chisholm et al., 2015; Min et al., 2015a,b; Zhu et al., 2015; Oni-Orisan et al., 2016; Kaushal et al., 2017; Pan et al., 2017; Chen et al., 2018; Hawasli et al., 2018; Ge et al., 2019; Karunakaran et al., 2020; Li et al., 2020a,b, 2023; Park et al., 2020; Black et al., 2021; Huynh et al., 2021; Nakanishi et al., 2021; Zheng et al., 2021, 2022; Guo et al., 2022; Kim et al., 2022; Vallesi et al., 2022; Wang et al., 2022; Mandloi et al., 2023) and several reports in nonhuman primates (Rao et al., 2014, 2016, 2022; Wu et al., 2017) and rodents (Seminowicz et al., 2012; Matsubayashi et al., 2018; Wu et al., 2020; Sanganahalli et al., 2021, 2022). The analysis of rs-fMRI in SCI seems to be in its infancy due to differences in the various rs-fMRI analysis methods (seed-based, ICA, graph theory, ReHo, ALFF, etc.) (Margulies et al., 2010; Lv et al., 2018), the timing of imaging, and degree of injury, and the lack of sufficient evidence based on animal experiments. For example, while reports of intra-insular network involvement in pain after SCI show similar tendencies (Li et al., 2020a,b; Huynh et al., 2021; Mandloi et al., 2023), reports of changes in motor cortex connectivity in relation to motor function are inconsistent (Hou et al., 2014; Min et al., 2015a,b; Zhu et al., 2015; Hou et al., 2016; Oni-Orisan et al., 2016; Rao et al., 2016; Kaushal et al., 2017; Pan et al., 2017; Hawasli et al., 2018; Matsubayashi et al., 2018; Li et al., 2020a,b; Park et al., 2020; Nakanishi et al., 2021; Kim et al., 2022; Wang et al., 2022). In the present study, network analysis revealed a shift in main hub function from the pyramidal/cortical to the extrapyramidal/subcortical system. Graph theory can withstand network analysis to calculate centrality, etc. because it sets up a large number of nodes to which functional meaning is assigned (Min et al., 2015a,b; Kaushal et al., 2017; Matsubayashi et al., 2018). Recently, network analysis has become possible without using thresholded binary data (Gopalsamy et al., 2023), and there is a possibility that raw serial rs-fMRI data can be adapted. More research using rs-fMRI in SCI should be conducted, especially in animal models.

In this study, we used a thoracic SCI model in which the lower limbs are mainly affected and confirmed that dynamic network changes occur in the motor cortex. In particular, the shift of the motor hub from the cortical to the subcortical system is consistent with a previous human clinical study (Kim et al., 2022). Since cortical hub function was not reduced in the transection-complete SCI model, this may reflect a successful transition to compensatory neural circuits. However, unlike humans, the rodent motor cortex has limited function in voluntary movements and is often lumped together with the “sensorimotor” system. Particularly in the lower limb, the role of the motor cortex is thought to be limited to movements in coordination with the upper limb since there is sufficient movement of the lower limb even after the corticospinal tract is severed, and the axonal terminals are located in the dorsal horn of the gray matter. Why, then, have changes occurred in such a large network? First, the cells of the rodent motor cortex have fibers that extend to the lumbar spinal cord, but there are also branches in the brainstem and cervical spinal cord (Lu et al., 2022), suggesting that the axonal transection affected the larger network. Second, although we could not subdivide the motor cortex because there were only 244 nodes in the entire brain, analysis by the motor cortex subtype might have revealed that the upper limb region is relatively preserved. Third, besides the classical anatomical structures that have been revealed by tracers and electrical organization, new aspects of the brain map have been reported (Graziano, 2016; Gordon et al., 2023). Namely, brain regions could be divided according to their functions, in addition to corresponding body parts. As mentioned above, because rodents have upper and lower limb coordination during walking, the limitation of lower limb capacity may affect the overall function of controlling gait and cause significant changes in the motor cortex. Further studies are needed to elucidate the cause of the large changes in the motor cortex network in rodents after lower limb impairment.

rs-fMRI can calculate many parameters and may be useful in understanding the pathophysiology of SCI. In this study, significant differences were observed at 7 and 14 weeks after injury. In rodent SCI models, spontaneous recovery of motor function reaches a plateau at 6 weeks (Supplementary Figure 3), which is considered the “chronic phase” (Shinozaki et al., 2011, 2014). In the brain, however, further changes have been observed from 7 to 14 weeks, with continuous changes from 1 to 14 weeks in macroscopic measures such as mean order centrality, and more fine changes in individual degree centrality. There are reports of changes in rs-fMRI parameters with rehabilitation after SCI (Chisholm et al., 2015; Rao et al., 2022). Even when if changes in motor function are not detected, pathophysiological indices such as rs-fMRI may be useful in selecting treatment modalities and determining treatment efficacy.

There are several limitations with this paper. First, it mainly uses the same incomplete injured thoracic spinal cord model and does not represent the causal relationship between pathophysiology and brain networks. Second, the data were converted into binary data for network analysis, but there is no clear standard for setting the threshold value, unlike other studies (Gerhard et al., 2011). We chose 0.1 as the threshold because when we increased the threshold above 0.10, information centrality could not be calculated (data not shown). Third, because this study was performed only on the pathophysiological untreated model, it did not consider the choice of treatment or the evaluation of treatment effects, which would be revealed by the protocols with treatment interventions at different time points after injury. Fourth, the intervals of the time series are different, so it is not possible to examine the amount of change and the time series in detail. Fifth, although nodes with edges to primary motor cortices were extracted for the study of locomotion, there may be nodes more universally related to locomotion in rodents. Finally, due to the facility where the MRI was performed, behavioral analysis was not performed in the same individuals, and tissue evaluation was not performed. In particular, the rs-fMRI connectivity data would have been strongly supported if tissue assessment had been performed using tracers.

Our results suggest that network analysis of rs-fMRI data may reflect the pathophysiology of SCI. In the future, network analysis is expected to be used to select treatment modalities for SCI and to evaluate the efficacy of treatment modalities through further evidence of rs-fMRI in SCI.

## Data availability statement

The original contributions presented in the study are included in the article/Supplementary material, further inquiries can be directed to the corresponding authors.

## Ethics statement

The animal study was approved by the Animal Study/Committee of the Central Institute for Experimental Animals (approval number: 14078). The study was conducted in accordance with the local legislation and institutional requirements.

## Author contributions

OT, KF, MN, and HO contributed to conception and design of the study. KM, MS, JH, YK, and NN performed surgeries and experiments, collected the data, and contributed to figure generation. MS performed the statistical analysis and wrote the sections of the manuscript. All authors read and approved the submitted version.

## Footnotes

^1^https://www.r-project.org/

^2^https://processing.org/

## Supplementary material

Supplementary Figure 1 3D schema of all nodes. Please refer supplementary Movie 1 for all directions.

Supplementary Figure 2 Spontaneous recovery and stability of the contused incomplete spinal cord injury model used in this study. An open field score (Basso Mouse Scale) was observed. Scores reached a plateau at 3 weeks; *N* = 4. Error bars are shown as SD.

Supplementary Figure 3 Changes over time of nodes with high centrality degree before or after injury. Error bars are shown as SEM.
