# Supplementary figures and images for: A shift of brain network hub after spinal cord injury
Source: Front Mol Neurosci. 2023 Oct 17;16:1245902. doi: 10.3389/fnmol.2023.1245902 (PMC10616864; doi:10.3389/fnmol.2023.1245902)

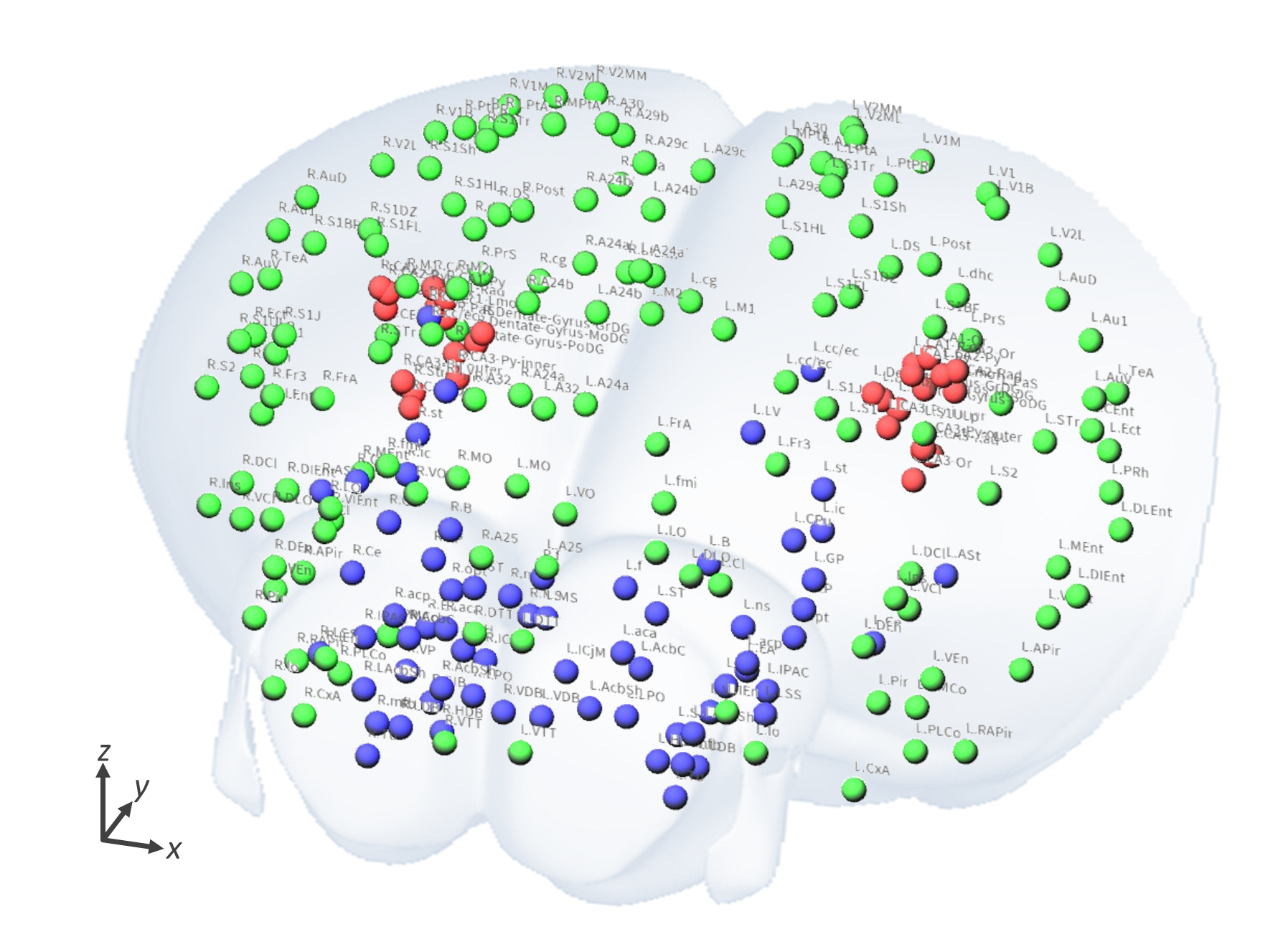

Supplement: Supplementary Figure 1 — 3D schema of all nodes. Please refer supplementary Movie 1 for all directions. [file Image_1.tif]

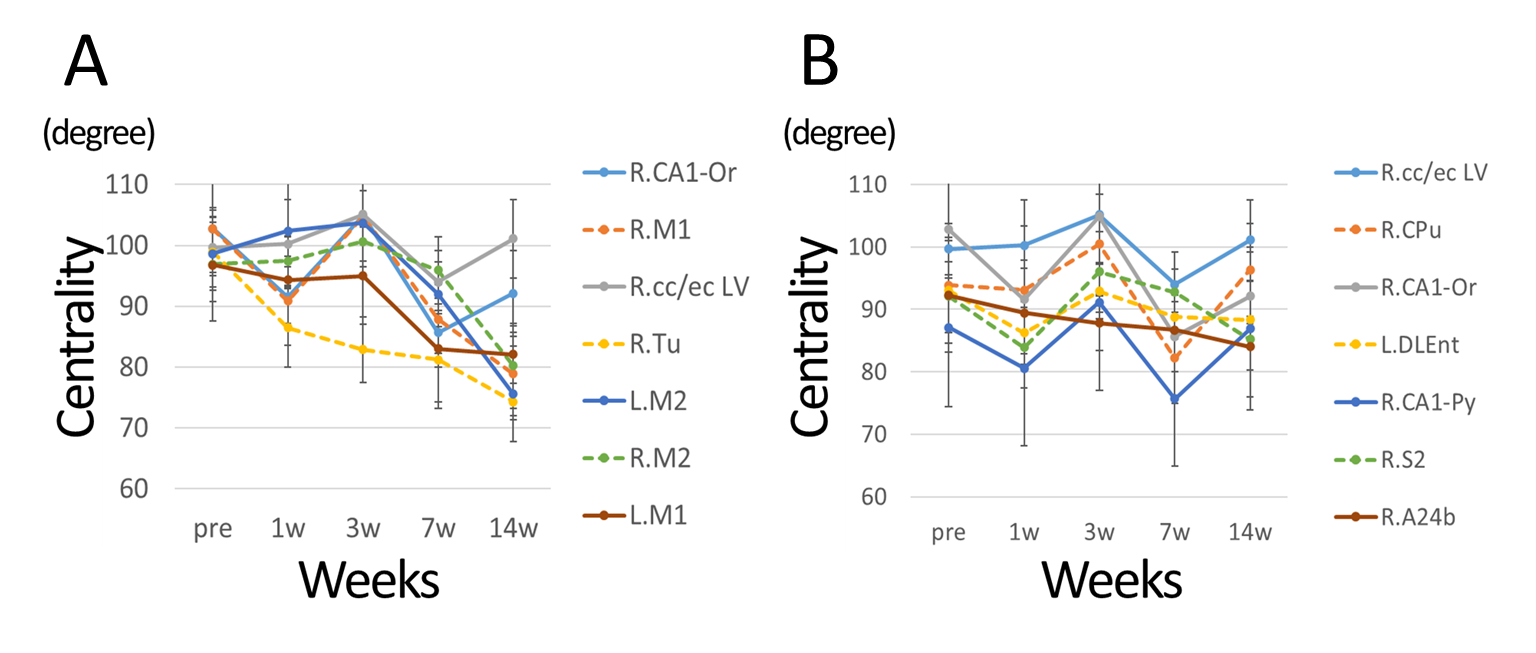

Supplement: Supplementary Figure 2 — Spontaneous recovery and stability of the contused incomplete spinal cord injury model used in this study. An open field score (Basso Mouse Scale) was observed. Scores reached a plateau at 3 weeks; N = 4. Error bars are shown as SD. [file Image_2.tif]

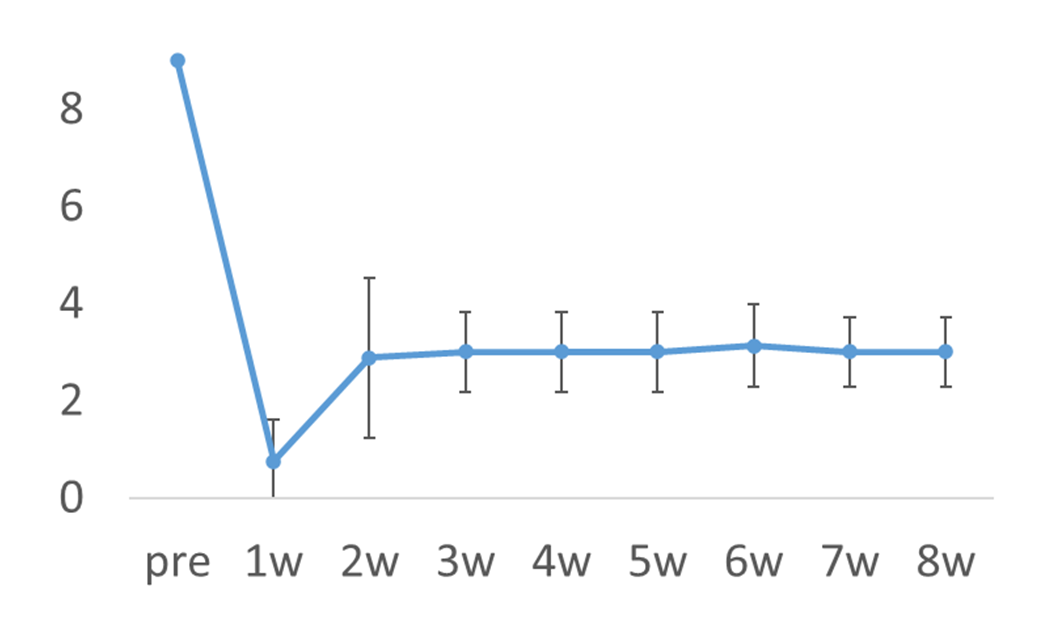

Supplement: Supplementary Figure 3 — Changes over time of nodes with high centrality degree before or after injury. Error bars are shown as SEM. [file Image_3.tif]
